# Smad4 and FoxH1 potentially interact to regulate *cyp19a1a* promoter in the ovary of ricefield eel (*Monopterus albus*)

**DOI:** 10.1186/s13293-024-00636-w

**Published:** 2024-07-30

**Authors:** Qiqi Chen, Deying Yang, Mingqiang Chen, Jinxin Xiong, Junjie Huang, Wenxiang Ding, Kuo Gao, Bolin Lai, Li Zheng, Ziting Tang, Mingwang Zhang, Taiming Yan, Zhi He

**Affiliations:** College of Animal Science and Technology, Sichuan Agricultural University, Chengdu, 611130 China

**Keywords:** Smad4, FoxH1, Ovary development, *Cyp19a1a* promoter, *Monopterus albus*

## Abstract

**Background:**

Cyp19a1a is a key enzyme in the pathway that converts androgens into estrogen and is regulated by TGF-β signaling. Smad4 and FoxH1 are downstream effectors of TGF-β signaling and may play important roles in ovarian development in *M. albus*.

**Methods:**

We investigated the expression pattern of the Smad4 and FoxH1 using qRT‒PCR and immunofluorescence, then tested the changes of *smad4* and *foxh1* by qRT‒PCR after ovary incubation with FSH in vitro, and analysed the regulation of *cyp19a1a* transcription by Smad4 and FoxH1 by dual-luciferase reporter assays.

**Results:**

We found that Smad4 encoded a putative protein of 449 amino acids and harbored the three conserved domains typical of this protein family. *Smad4* and *foxh1* exhibited similar expression patterns during ovarian development and after FSH incubation, with Pearson’s coefficients of 0.873 and 0.63–0.81, respectively. Furthermore, Smad4, FoxH1 and Cyp19a1a colocalized in the granulosa cells and theca cells of ovaries during the mid-to-late vitellogenic stage. Smad4 repressed *cyp19a1a* activity via SBE1 (− 1372/−1364) and SBE2 (− 415/−407) in the *cyp19a1a* promoter, whereas mutating SBE1 or SBE2 restored *cyp19a1a* promoter activity. Co-overexpression of Smad4 and FoxH1 significantly reduced *cyp19a1a* promoter activity.

**Conclusions:**

This study provides new insights into the potential functions of transcription factors Smad4 and FoxH1 in ovarian development and the transcriptional regulation mechanism of *cyp19a1a* in *M. albus*, which will reveal Smad4/FoxH1-mediated TGF-β signaling in reproduction and the regulation of the *cyp19a1a.*

**Plain English summary:**

Aromatase, encoded by *cyp19a1a*, is involved in ovarian development and plays an important role in the quality of eggs, as well the sex ratio, of the teleost fish, *M. albus.* The research on the transcriptional regulation of *cyp19a1a* has contributed to the understanding of its role in ovarian development. In previous study, it was shown that FoxH1 inhibits *cyp19a1a* transcription. In the present study, Smad4 was confirmed as a *cyp19a1a* transcriptional repressor and Smad4 may also coordinate with FoxH1 to repress *cyp19a1a* transcription. At present, we provide a new perspective for the transcriptional regulation of *cyp19a1a* by transcription factors Smad4 and FoxH1 in teleost fish ovary. In the future, the regulatory networks of Smad4 and FoxH1 will be further studied and the gene editing technology will be applied to screen specific regulatory factors of cyp191a1a gene, so as to alter the female cycle and modulate the sex ratio of the eggs production.

**Supplementary Information:**

The online version contains supplementary material available at 10.1186/s13293-024-00636-w.

## Highlights

- *Smad4* and *foxh1* were closely correlated with ovarian development, and Pearson’s coefficient was 0.873.
- FSH incubation stimulated the expression of *smad4* and *foxh1* in the ovaries.
- Smad4, FoxH1 and Cyp19a1a colocalized in the granulosa cells and theca cells of ovaries.
- Smad4 and FoxH1 potentially synergistically suppress the activity of *cyp19a1a* promoter.

**Supplementary Information:**

The online version contains supplementary material available at 10.1186/s13293-024-00636-w.

## Background

The transforming growth factor-β (TGF‐β) signaling pathway is involved in multiple processes associated with gonad development, including folliculogenesis, steroid hormone production, granulosa cell (GC) proliferation and feedback regulation between the pituitary and gonad [1–4]. SMADs (SMA and mother against decapentaplegic (MAD)-related proteins) are intracellular transducers of TGF-β superfamily signaling. Smad4, the only common mediator SMAD (Co-Smad: Smad4), is the central mediator of the canonical TGF-β signaling pathway. SMAD4 interacts with receptor‐activated SMAD proteins SMAD1/5/8 and SMAD2/3 to form the Smad complex that finally translocates into the nucleus to regulate the transcription of target genes [5]. Several studies shown that Smad2/4, Smad3/4 or Smad2/3/4 can mediate TGF-β signaling to stimulate aromatase expression in GCs, suggesting that Smad4 plays an integral role in this process, which was blocked when Smad4 was knocked down [6–9]. Similarly, knockdown of Smad4 inhibited FSH (Follicle stimulating hormone)‐induced aromatase expression in GCs [10]. Furthermore, luciferase and chromatin immunoprecipitation (ChIP) assays confirmed that Smad4 positively regulates the transcriptional activity of the porcine CYP19A1 gene (encodes aromatase, the key enzyme for estrogen biosynthesis) by directly binding to its promoter [11]. These results clearly indicate that TGF-β/Smad4 signaling regulates the expression of aromatase.

Smad4 homologs have been identified in mammals and teleosts and share a typical domain structure with Smad2/3, namely, the Mad homology 1 (MH1) domain and MH2 domains, but possess no Ser-Ser-X-Ser (SSXS) phosphorylation motif at the carboxy terminus [11]. In mammals, SMAD4 exhibits stage-specific expression during follicle development. Smad4 presents a differential expression pattern in different stages of follicle development and was expressed in multiple types of ovarian follicles, such as mainly localization in the cytoplasm of oocytes, GCs and theca cells (TCs) in human [12], pig [13], rat [14, 15], mouse [16], fetal baboon [17] and European hedgehog [18]. Moreover, SMAD4 is absolutely required for normal FSH synthesis in both male and female mice to regulate gonadal function [19]. However, specific deletion of SMAD4 in GCs of preovulatory follicles in Cyp19-Cre mice led to follicular atresia [20]. In contrast, knockout of SMAD4 in Amhr2-Cre mice caused premature luteinization of GCs and eventually premature ovarian failure [21]. Smad4 was expressed in the oocytes and follicle layers of zebrafish but was higher in the oocytes [22]. However, in yellow catfish, *smad4* mRNA levels were the highest in the testis, followed by the liver and ovary [23]. The above results show that Smad4 may play a crucial role in female reproduction, including ovarian development, gonadotropin synthesis and GCs survival.

The ricefield eel *Monopterus albus* is a protogynous hermaphrodite fish that naturally undergoes a sex change from a functional female to a male through an intersexual phase of gonad development [24, 25]. Previously, some members of the TGF-β superfamily were detected in *M. albus*, including Gdf9 [26], Gsdf [27], Bmpr2a [28] and Smad2 [29], which play important roles in gonadal development. SMAD4 also was shown to enhance the expression of *CYP19A1* gene in mammals [6] and up-regulate porcine *CYP19A1* promoter activities in vitro [8]. Our previous study found that FoxH1 inhibited *cyp19a1a* transcription [30]. Smad4 and FoxH1 complexes regulate target genes in response to TGF-β signals. However, little is known about the roles of Smad4 in *M. albus* gonadal development. To shed light on the role of Smad4 and FoxH1 in gonadal development and in the regulation of *cyp19a1a* promoter activities, the spatiotemporal expression patterns of Smad4 and regulation by hCG (human chorionic gonadotropin) and FSH in vitro were analyzed. Then, the colocalization of Smad4, FoxH1 and Cyp19a1a in the ovary was also determined. The activity and sites of Smad4 binding to the *cyp19a1a* promoter were evaluated and screened. This study provides novel insights into Smad4/FoxH1-mediated TGF-β signaling in reproduction and the regulation of the *cyp19a1a* promoter in teleosts.

## Materials and methods

### Sample collection and preparation

Healthy *M. albus* (*n* = 100, body length = 34.93 ± 4.52 cm and body weight = 37.99 ± 21.88 g) were obtained from a local market in Chengdu, Sichuan. Fish were fasted for 24 h and then anesthetized with 0.02% tricaine buffer (80 µg/L) (Sigma, LA, USA) for 10 min. The half of the gonad, pituitary gland, eye, heart, kidney, intestine, spleen, muscle and blood, were collected and immediately stored in liquid nitrogen at -80 °C. A portion of the fresh gonads was immediately fixed with Bouin’s solution for 24 h and embedded in paraffin. Sections were serially cut at a thickness of 5 μm using a slicer (Leica, Nussloch, Germany) and stained with hematoxylin/eosin. The histological classification of the gonad as primary growth (PG), previtellogenic (PV), early vitellogenic (EV), mid-to-late vitellogenic (MLV), or mature (OM) stage has been described previously [29]. All procedures and investigations involving animals were approved and performed in accordance with the guidelines of the ethics committee (Approval No. 20,190,031).

### RNA isolation

Total RNA from all samples was isolated using TRIzol (Invitrogen, CA, USA) according to the manufacturer’s instructions. RNA quality and quantity were evaluated by measuring the absorbance at 260 and 280 nm and by performing 1.0% agarose gel electrophoresis. The genomic DNA was eliminated by treatment with gDNA Eraser (Trans Biotech, Beijing, China) before reverse transcription was performed.

### Cloning of *smad4* cDNA

The cDNAs were transcribed from the total RNA (1.0 µg) of ovaries using the RevertAid First-strand cDNA Synthesis Kit (Thermo, IN, USA) according to the manufacturer’s instructions. The cDNA quality was verified by the successful amplification of *ef1α* and *rpl17* [31]. Specific primers to clone *smad4* (*smad4*-F1, *smad4*-R1, *smad4*-F2 and *smad4*-R2, in Table S1) were designed based on the coding sequence of the *M. albus* genome (Accession No: 109,961,575).

PCR was performed in 10 µL reactions containing 0.5 µLof each primer (10 µM), 5.0 µL of 2× Taq Master Mix (Vazyme, Nanjing, China), 3 µL of ddH_2_O and 1 µL of gonad cDNA template. The PCR conditions were as follows: an initial 3 min denaturation at 95 ℃, 32 amplification cycles of 0.5 min at 95 ℃, 0.5 min at 55 ℃, and 1.5 min at 72 ℃, followed by a final extension for 30 min at 72℃. All target products were ligated into the pMD19-T vector (TaKaRa, Dalian, China) and sequenced by TsingKE Biological Technology Company Limited (Chengdu, Sichuan, China).

### Sequence analysis

The sequence of the *smad4* gene was confirmed by the web-based tool BLAST (https://blast.ncbi.nlm.nih.gov/Blast.cgi). The DNA sequence was spliced and translated by the DNAMAN software translation tool, and the web-based tool CSS-Palm was used to predict L-acetylation sites. A phylogenetic tree was constructed based on the deduced amino acid sequences using the neighbor-joining method with bootstrap values calculated from 1000 replicates in the MEGA 11.0 software package. The protein domains of Smad4 were predicted by the TMHMM web server v2.0 and SMART version 9.

### Quantitative real-time polymerase chain reaction (RT-qPCR) analysis

cDNA was obtained from gonadal tissues. RT‒qPCR was performed according to a previous study [25], and cDNA quality was verified by successful amplification of *ef1α* and *rpl17*. The relative expression levels of target genes were calculated through the double internal reference approach. Target gene expression was calculated with the equation

$${C_{target}}{\,_{gene}}/\sqrt {{{\rm{C}}_{{\rm{ef1}}}} \times {{\rm{C}}_{{\rm{rpl17}}}}}$$

The primers for qRT‒PCR are listed in Supplementary Table S1.

### Immunofluorescence

Immunofluorescence was used to evaluate Smad4, FoxH1 and Cyp19a1a localization in paraffin-embedded ovarian tissues at the EV and MLV stages. Briefly, ovarian sections were deparaffinized, rehydrated, and subjected to high-temperature (95–98 ℃) antigen retrieval for 10 min with EDTA (pH 8.0). Then, the sections were blocked in 3% BSA for 30 min at room temperature and incubated with primary antibodies overnight at 4 ℃. The primary antibodies used were Smad4 (GeneTex, GTX112980, 1:1000), FoxH1 (Genetex, GTX17182, 1:1000) and Cyp19a1a (Genetex, GTX18995, 1:1000). Subsequently, the sections were incubated with fluorophore-conjugated goat anti-rabbit secondary antibodies (Servicebio, GB23303, 1:2000) for 2 h at 37 ℃. Finally, the sections were coverslipped using anti-fade fluorescent mounting medium (Servicebio, G1221-5ML), imaged using a Pannoramic 250 fully automated digital scanning microscope (3DHISTECH) and observed with the CaseViewer application (3DHISTECH).

### Expression patterns of smad4 in ovaries after incubation with hCG and FSH in vitro

Ovaries of female *M. albus* at the MLV stage were washed and dissected in Leibovitz L-15 medium (Gibco, Massachusetts, USA) on ice. Ovarian tissues (50–100 mg) were placed in 24-well tissue culture dishes in 1 mL of Leibovitz L-15 medium (0.1 U/mL penicillin and 0.1 mg/ml streptomycin) with FSH (Sigma-Aldrich, 0.05, 1.0, and 5.0 ng/mL), hCG (Sigma-Aldrich, 10, 50, and 100 IU/mL), or saline solution (control group) and then incubated at 28 ℃ for 1, 2, 4, and 10 h.

### Plasmids construction and luciferase assay

The *cyp19a1a* promoter with a double luciferase reporter vector and FoxH1 overexpression vector were constructed previously [30]. For Smad4 overexpression vector construction, the full-length *smad4* coding sequence (1533 bp) of *M. albus* was amplified, double-digested with *NheI* and *EcoRI* and then cloned and inserted into the pcDNA3.1 vector. Additionally, mutant vectors were constructed by using a TaKaRa Mutant BEST Kit (#R401, TaKaRa, Beijing, China). The sequences of wild-type and mutant Smad binding elements (SBEs) are shown in Table S2. All recombinant plasmids were constructed by Bioengineering (Shanghai) Co., Ltd. and verified by Sanger sequencing.

For luciferase activity detection, after transfection for 48 h, the cells were harvested, and their lysates were collected for dual-luciferase analysis with a Dual-Luciferase Reporter Assay System (#E1910, Promega, Madison, USA) following the kit’s manual. The GloMax detection system (Promega) was used to measure firefly and Renilla luciferase activity in cell lysates.

### Statistical analysis

Data are presented as the means ± SEMs. The statistical significance of differences was analyzed with one-way ANOVA followed by Duncan’s multiple comparison test using SPSS 22.0 software (SPSS, Inc., NY, USA). Data correlations were analyzed using Pearson correlation analysis with SPSS 17.0 software. Significance was set at *p* < 0.05.

## Results

### Sequence analysis of *M. albus smad4*

**Fig. 1:**
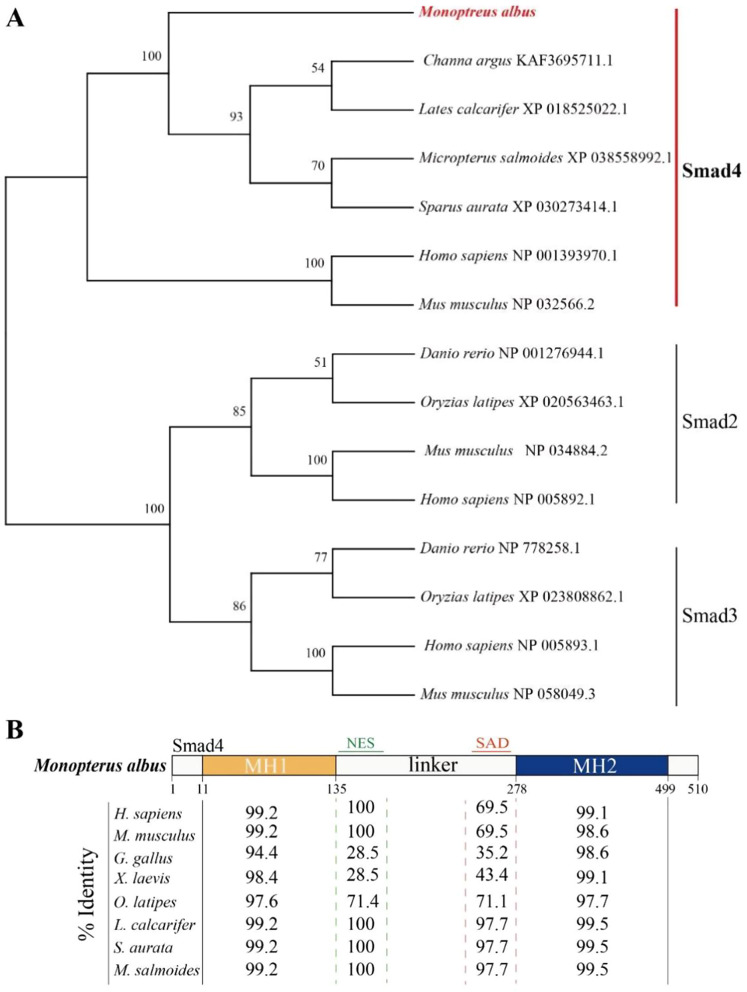
Phylogenetic analysis and domain characteristics of Smad4 in the ricefield eel *Monopterus albus*. **A** Neighbor-joining phylogenetic trees of Smad4. The numbers at the nodes are the bootstrap proportions. Other vertebrate protein sequences were downloaded from the NCBI database (accession numbers in parentheses). **B** The characteristic Smad4 domains are conserved in ricefield eel orthologs. Numbers represent the percentage identity of the predicted protein sequence shared with other Smad4 orthologs (*Homo sapiens*, NP_001393970.1; *Mus musculus*, NP_032566.2; *Gallus gallus*, XP_040508511.1; *Xenopus laevis*, XP_002934485.1; *Oryzias latipes*, XP_004074821.1; *Lates calcarifer*, XP 01 8525022.1; *Sparus curate*, XP 030273414.1 *Micropterus salmoides*, XP 038558992.1)

The open reading frame (ORF) of *M. albus smad4* was 1533 base pairs (bp) in length, consisting of 9 exons and encoding a putative protein of 510 amino acids (aa) (Supplementary Fig. S1). Phylogenetic analysis also indicated that *M. albus* Smad4 had high sequence identity with homologs in other teleosts (Fig. 1A). Multiple sequence alignment showed that the sequence homology of *smad4* genes ranged from 69.73 to 84% (Fig. 1B). Similar to those of other vertebrates, the predicted *M. albus* Smad4 possessed an MH1 domain at the N-terminus, an MH1 domain at the C-terminus, a Smad4 activation domain (SAD), a DNA binding domain (DBD), and nuclear localization and export signals (NLS and NES, respectively) (Fig. 1B).

### Expression patterns of *smad4* in tissues and during ovarian development

**Fig. 2:**
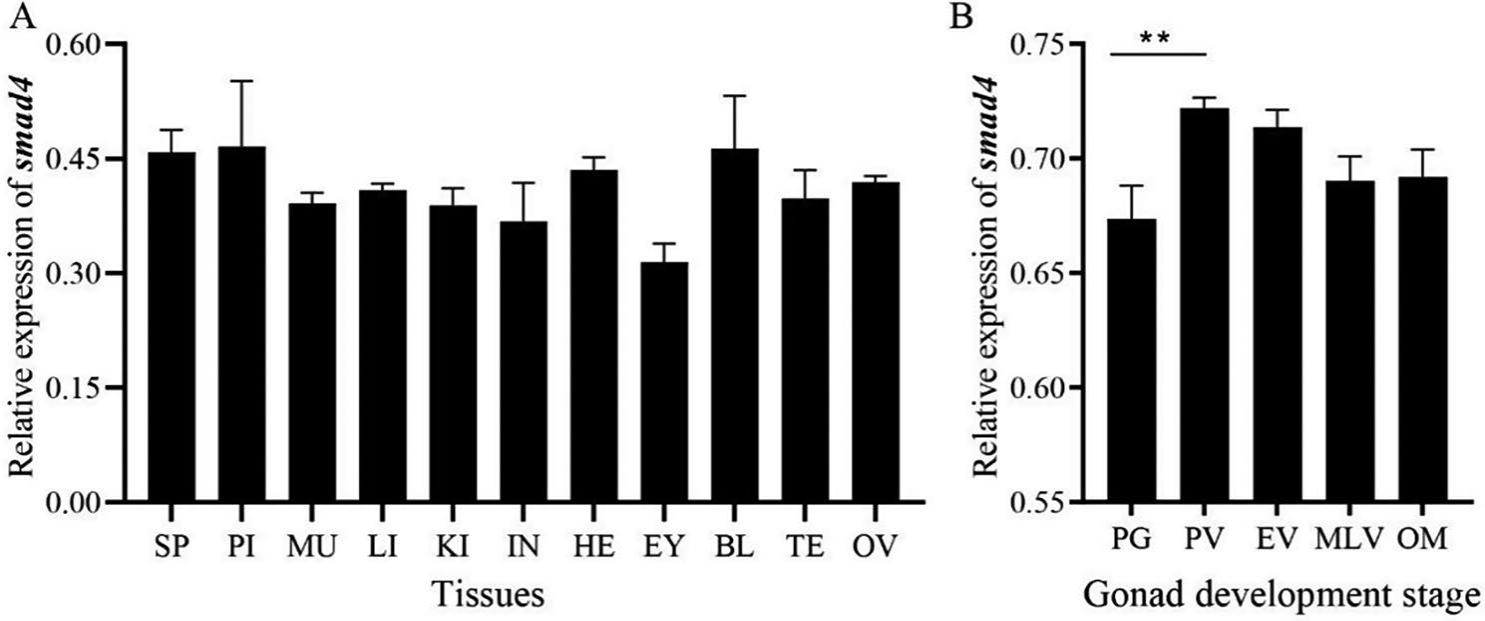
Expression level of *smad4* in different tissues and during ovarian development in *Monopterus albus*. **A** Relative mRNA levels of *smad4* in the tissues. **B** Relative *smad4* mRNA levels during ovarian development. *BL* Blood, *EY* Eye, *HE* Heart, *IN* Intestines, *KI* Kidney, *LI* Liver, *MU* Muscle, *PI* Pituitary, *SP* Spleen, *TE* Testis, *OV* Ovary. The results are presented as the means ± SEMs (*n* = 4). **p < 0.01

**Fig. 3:**
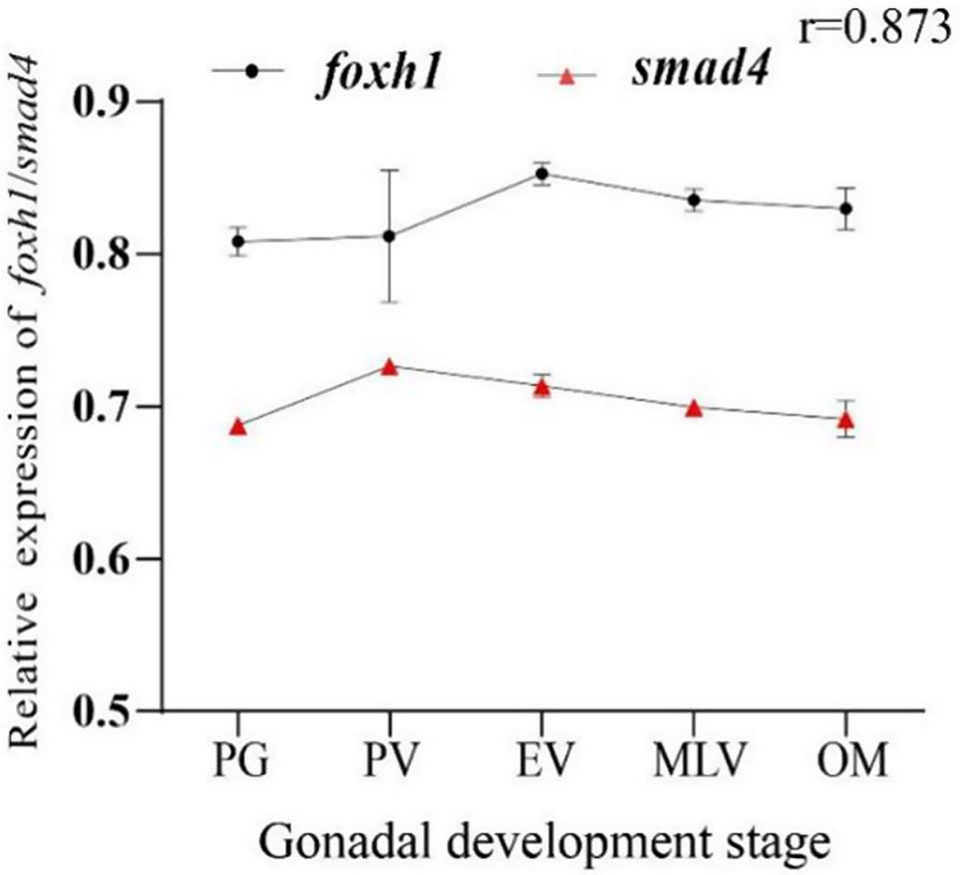
The mRNA expression profiles of *smad4* and *foxh1* during ovarian development. The results are presented as the means ± SEMs (*n* = 4). The Pearson’s coefficient between *smad4* and *foxh1* was 0.873 (*p* < 0.01**)**

Smad4 mRNA was broadly expressed in all examined tissues, but its mRNA levels differed among tissues (*p* < 0.05). The highest mRNA expression level of *smad4* was detected in the pituitary, followed by the blood, and the lowest was detected in the eye. There was no significant difference in the expression level of *smad4* between the testis and ovary (*p* > 0.05) (Fig. 2A). During ovarian development, *smad4* mRNA levels were highest at the PV stage (*p* < 0.01) and then gradually decreased with further development (Fig. 2B). *Smad4* and *foxh1* had similar expression patterns (Fig. 3), and their Pearson’s coefficient was 0.873 (*p* < 0.01).

### Expression of *smad4* in the ovary after incubation with FSH and hCG

**Fig. 4:**
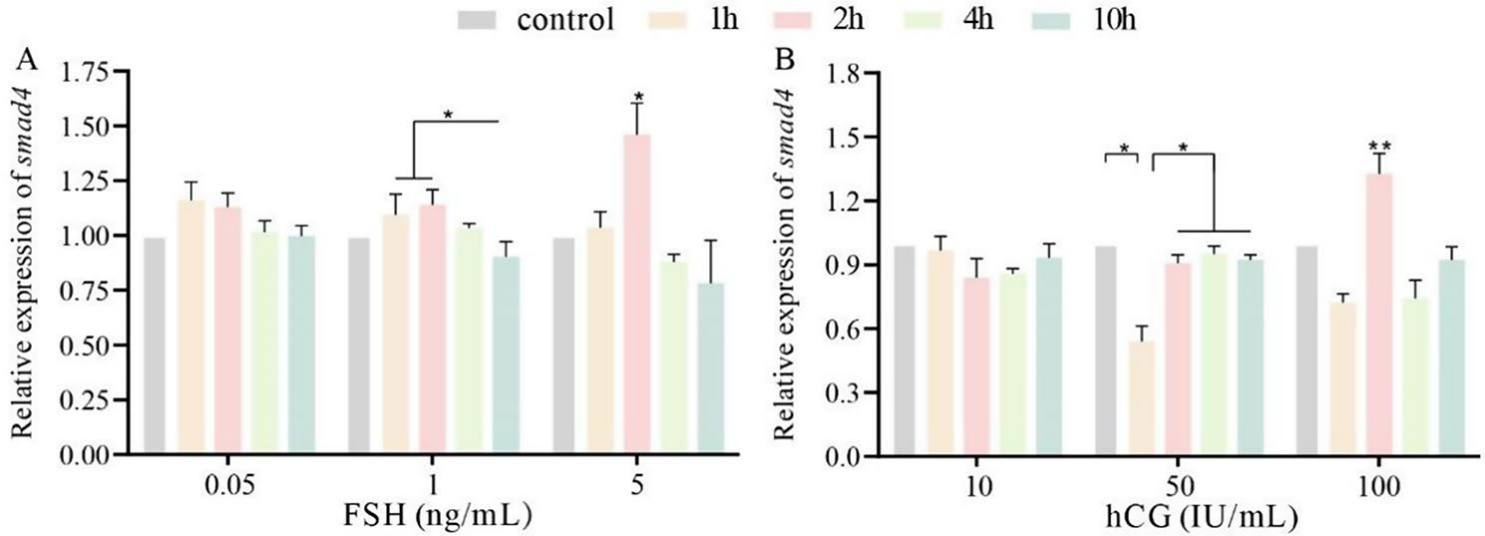
Regulation of *smad4* expression in the ovary by FSH and hCG in vitro. **A** Expression level of *smad4* after FSH incubation. **B** Expression level of *smad4* after hCG incubation. The results are presented as the means ± SEMs (*n* = 5). *FSH* Follicle stimulating hormone, *hCG* Human chorionic gonadotropin, *p < 0.05

FSH and hCG had different stimulatory effects on *smad4* expression levels. In the 0.05 ng/L FSH group, *smad4* expression levels did not change significantly at different treatment times (*p* > 0.05) but tended to increase at 1 h and 2 h and then decrease at 4 h. In the 1 and 5 ng/mL FSH groups, the expression levels of *smad4* increased and peaked at 2 h, followed by a significant decrease (*p* < 0.05) (Fig. 4A). After hCG incubation, the expression level of *smad4* was the lowest at 1 h and significantly increased at 2 h (*p* < 0.05) in the 50 IU/mL and 100 IU/mL groups, but not in the 10 IU/mL group. In addition, the expression level of *smad4* was not different from that in the control group after 10 h of incubation with different concentrations of hCG (Fig. 4B).

Pearson’s correlation coefficient showed strong positive relationships between *smad4* and *foxh1* after FSH incubation (Table 1). After 2 h of incubation with FSH, the correlation coefficient between *foxh1* and *smad4* was 0.97 (*p* < 0.01). The correlations between *smad4* and *foxh1* were highly positive, with coefficients ranging from 0.63 to 0.81 in the presence of different concentrations of FSH.

**Table 1:** Correlation analysis between *foxh1* and *smad4* after FSH incubation in vitro

		Correlation coefficient
Time	1 h	0.67^**^
	2 h	0.97^**^
	4 h	0.80^**^
	10 h	0.30
Concentration of FSH	0.05 ng/L	0.75^**^
	5 ng/L	0.81^**^
	1 ng/L	0.63^**^

*Notes FSH* follicle stimulating hormone, **p < 0.01

### Colocalization of Smad4, FoxH1 and Cyp19a1a in EV-stage *M. albus* ovaries

**Fig. 5:**
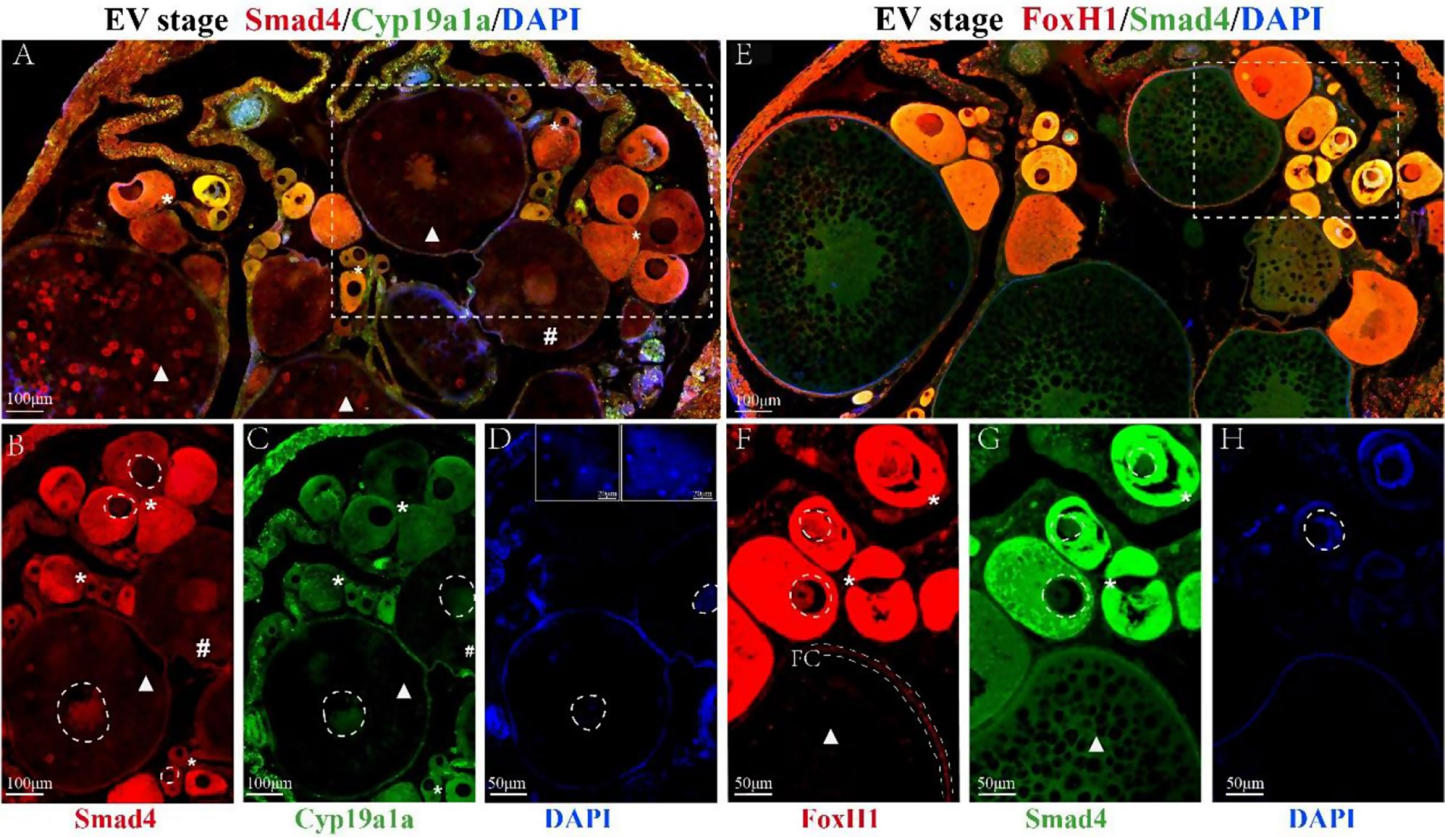
Immunofluorescence localization of Smad4, FoxH1 and Cyp19a1a signals in the early vitellogenic (EV) stage *M. albus* ovary. **A** Colocalization of Smad4 and Cyp19a1a immunostaining. **B** Smad4 immunostaining. **C** Cyp19a1a immunostaining. **D**,** H** Nuclei are labeled with DAPI (blue). **E** Colocalization of Smad4 and FoxH1 immunostaining. **F** FoxH1 immunostaining. **G** Smad4 immunostaining. Asterisk (*), *PGOs* Primary growth oocytes, pound sign (#), *CAOs* Cortical alveoli stage oocytes, triangle (△), *EVOs* Early vitellogenic-stage oocytes, *FC* Follicular cells

**Fig. 6:**
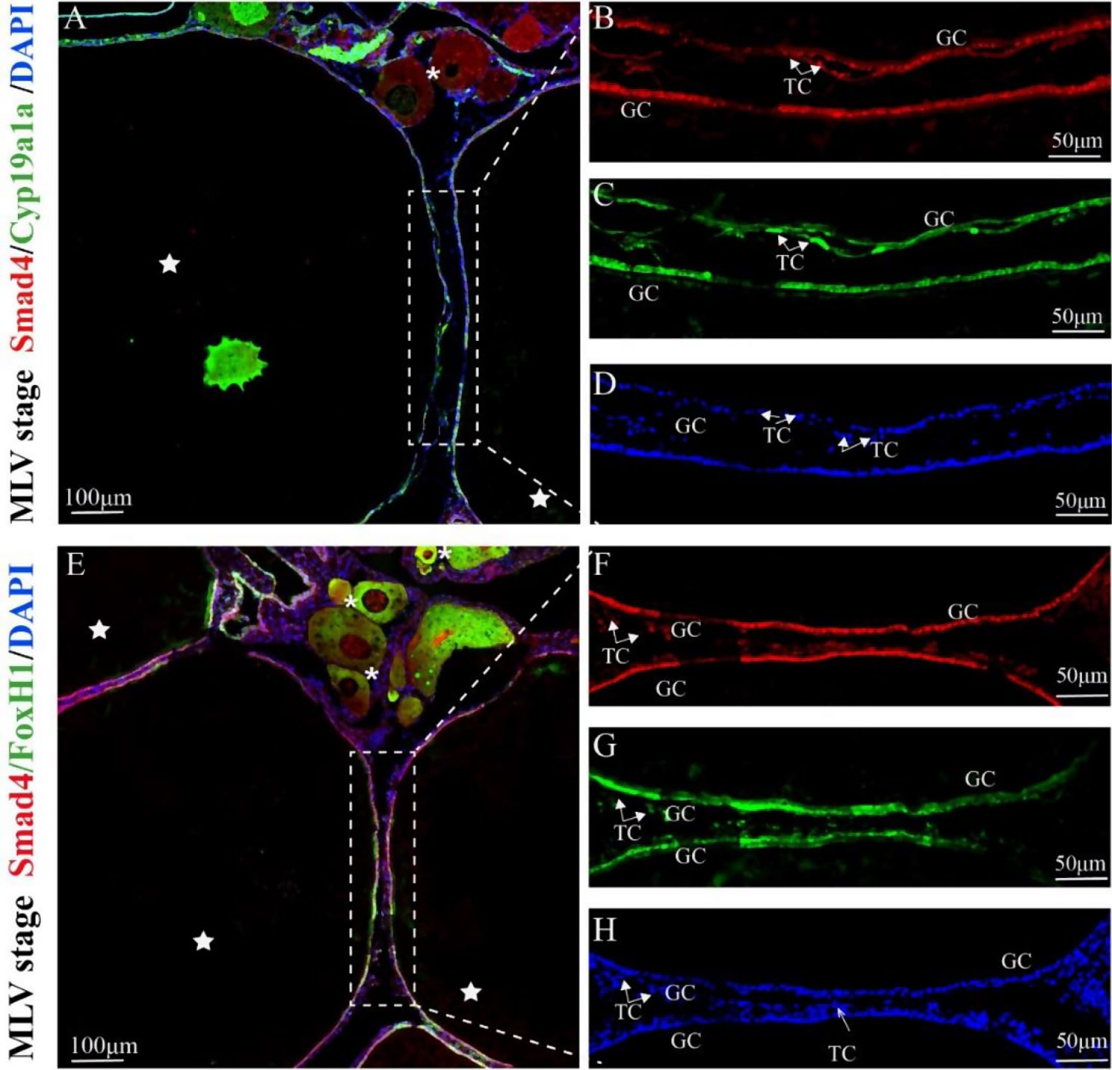
Immunofluorescence localization of Smad4, FoxH1 and Cyp19a1a signals in the mid-to-late vitellogenic (MLV) stage *M. albus* ovary. **A** Colocalization of Smad4 and Cyp19a1a immunostaining. **B** Smad4 immunostaining. **C** Cyp19a1a immunostaining. **D**,** H** Nuclei are labeled with DAPI (blue). **E** Colocalization of Smad4 and FoxH1 immunostaining. **F** FoxH1 immunostaining. **G** Smad4 immunostaining. Asterisk (*), *PGOs* Primary growth oocytes, pentagram (☆), *MLVOs* Middle- to late-vitellogenic-stage oocytes, *GC* Granulosa cells, *TC* Theca cells

The subcellular localization and colocalization of Smad4/Cyp19a1a and Smad4/FoxH1 in the EV and MLV stage ovaries were examined with immunofluorescence (Figs. 5 and 6). Smad4 was expressed predominantly in the nuclei and cytoplasm of primary growth oocytes (PGOs) and early vitellogenic stage oocytes (EVOs), but also in the follicular cells (Fig. 5B). Cyp19a1a was localized in the cytoplasm of PGOs, the cytoplasm of cortical alveoli stage oocytes (CAOs) and the nuclei of EVOs (Fig. 5C). FoxH1 was present in the nuclei and cytoplasm of PGOs and the follicular cells of EV follicles (Fig. 5F). In EV follicles, Smad4/Cyp19a1a were colocalized in the cytoplasm of PGOs and the nuclei of EVOs, and Smad4/FoxH1 were colocalized in the nuclei and cytoplasm of PGOs.

Cyp19a1a was expressed mainly in the oocytes nuclei, GCs and TCs of MLV stage follicles. (Fig. 6C). Furthermore, the fluorescence signal of Cyp19a1a was stronger than that of FoxH1 and Smad4 in TCs. In the MLV follicles, Smad4/Cyp19a1a and Smad4/FoxH1 were colocalized in the GCs and TCs (Fig. 6A, E).

### Repression of *cyp19a1a* promoter by Smad4 via the SBE site in vitro

**Fig. 7:**
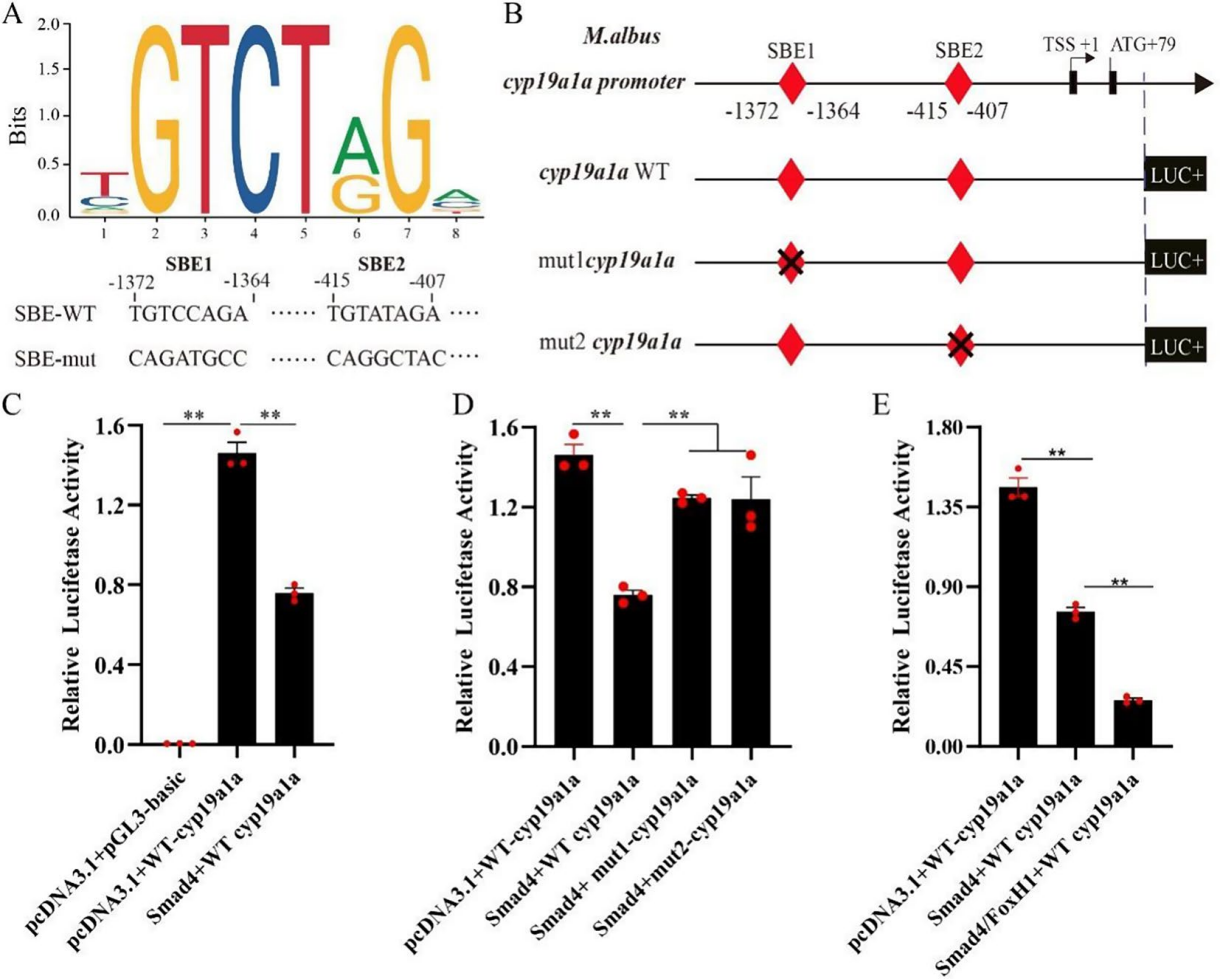
SMAD4 acted as a transcription factor and induced *cyp19a1a* transcription. **A** Consensus sequence of the Smad4 binding site based on the JASPAR database and Smad4 binding site sequence on the promoter of *cyp19a1a*. mut indicates SBE mutation. **B** Schematic showing that different loci of the *cyp19a1a* promoter were cloned and inserted into the pGL3 vector. Potential SBEs are indicated by red diamonds, and the transcription start site (TSS) was denoted as + 1. **C** The activity of *cyp19a1a* luciferase reporters in CHO cells with or without Smad4 overexpression was measured. **D** The activity of wild-type and mutant *cyp19a1a* luciferase reporters in CHO cells overexpressing Smad4 was measured. **E** The activities of wild-type *cyp19a1a* luciferase reporters in CHO cells overexpressing Smad4 and FoxH1 were measured. The results are presented as the means ± SEMs (*n* = 3). *p < 0.05; **p < 0.01

A previous study revealed that the *cyp19a1a* gene of *M. albus* has one TSS located 53 nt upstream of the start codon (ATG) [32] (Fig. 7). JASPAP analysis revealed two predicted Smad4 binding sites (SBEs), namely, SBE1 (− 1372/−1364) and SBE2 (− 415/−407). Luciferase activity analysis revealed that Smad4 overexpression significantly decreased the promoter activity of *cyp19a1a* (*p* < 0.01) (Fig. 7C). After SBE1 or SBE2 was mutated, the promoter activity of *cyp19a1a* was significantly elevated (*p* < 0.01). Compared with the overexpression of Smad4 alone, the co-overexpression of Smad4 and FoxH1 significantly reduced the promoter activity of *cyp19a1a* (*p* < 0.05) (Fig. 7E).

## Discussion

Smad4, the global partner of all receptor-regulated Smad proteins, belongs to the Smad family and is conserved across vertebrates [33]. In the present study, the ORF sequences of *smad4* were obtained from the gonads of *M. albus*, and their sequence features were analyzed. The results showed that the protein sequences of *M. albus* Smad4 had domains similar to those of mammals [34] and other teleosts [23], such as the N-terminal MH1 domain, C-terminal MH2 domain, SAD domain, DNA binding motif, NLS domain and NES domain. These data suggest that the functions of the Smad4 protein are similar between teleosts and mammals. In the present study, *smad4* mRNA levels were the highest in the pituitary, but there was no significant variation in the ovary and testis, consistent with previous findings in goldfish [35] and nile tilapia [36], suggesting that *smad4* may play key role in the regulation of brain–pituitary–gonad axis. Smad4 was widely distributed in all developmental stages of the ovary in *M. albus*, which was consistent with the results of studies in rat [15], pigs [13] and European hedgehog [18]. Furthermore, *smad4* and *foxh1* had similar expression patterns during ovary development, with a Pearson’s correlation coefficient of 0.873. These results suggested that *smad4* and *foxh1* may play crucial roles in folliculogenesis and ovary development.

The SMAD4 signaling pathway regulates GCs development and physiological function. In rat [15], pig [13], European hedgehog [18] and fetal baboon [17], Smad4 protein is mainly located in oocytes, GCs and TCs and diffusely distributed in the interstitial cells surrounding the follicle. SMAD4 silencing promoted GCs apoptosis [37, 38] and downregulated the expression levels of steroidogenesis-related genes including STAR, CYP19A1 and HSD3B [10, 39]. Furthermore, SMAD4 knockdown significantly inhibited FSH-induced estradiol production in GCs [39]. Aromatase, a key enzyme of estradiol synthesis and a marker of GCs differentiation, is essential for ovarian development and reproduction [40]. In the present study, Cyp19a1a was specifically expressed in GCs and TCs but not in oocytes, which was consistent with results from zebrafish [41]. Furthermore, Smad4/FoxH1, Smad4/Cyp19a1a and FoxH1/Cyp19a1a were colocalized to GCs and TCs, suggesting a possible regulation of the *cyp19a1a* gene by Smad4 and FoxH1 in *M. albus*.

Gonadotropins are essential regulators of gonadal function and fertility [42]. Smad4 was absolutely required for normal FSH synthesis in both male and female mice [19]. In mouse gonadotropes, deletion of *Smad4* led to FSH deficiency and female subfertility [43]. In pigs, knockdown of *SMAD4* significantly inhibited FSH-induced GC proliferation and estradiol production [10]. In addition, the levels of Smad4 mRNA and protein significantly increased after FSH injection [16]. In the present study, FSH stimulated *smad4* expression. In the 1 and 5 ng/mL FSH groups, the expression levels of *smad4* had a similar pattern of change, in which *smad4* levels were increased and peaked at 2 h and then recovered to the control level. Previous studies have shown that FSH stimulates *foxh1* expression but that this stimulation weakens with time [30]. After FSH incubation, *smad4* and *foxh1* had consistent expression patterns. Pearson’s relationships between *smad4* and *foxh1*. These results reveal that *smad4* is regulated by FSH and that *smad4*/*foxh1* may play a role in FSH-regulated ovarian developmental networks.

SMAD4 is a transcription factor that binds to the SBEs of target gene promoters via the N-terminal MH1 domain [44, 45]. Knockout of *SMAD4* in porcine granule cells significantly inhibited FSH-induced *CYP19A1* mRNA elevation and estradiol production via the BMP15→ TGFβRII→ SMAD4→ FSHR signaling pathways [10, 46]. The TGFβ1→ SMAD2/4→ CYP19A1 axis regulates aromatase expression and estradiol synthesis in human luteal GCs, and *CYP19A1* expression is inhibited when *SMAD4* is knocked down [6]. In addition, SMAD4 promoted *CYP19A1* transcription by directly binding to its promoter [8]. Transcription factors may have the species-specific regulatory mechanisms for the same target genes. Dmrt1 at doses from 10 to 250 ng repressed basal as well as Ad4BP/SF-1(NR5A1)-activated *cyp19a1a* transcription in *Oreochromis niloticus* [47]. Dmrt1a did not affect the *cyp19a1a* promoter activity at lower doses (less than 50 ng) but upregulated *cyp19a1a* transcription at higher doses (100–200 ng) in *M. albus*. Dmrt1 also reduced the Nr5a1a-induction of *cyp191a* transcription in *M. albus* at lower doses and this inhibition disappeared at higher doses from 50 to 200 ng [48]. In the present study, Smad4 and FoxH1 were colocalized in GCs and TCs. In addition, two Smad4-binding sites were predicted in the *cyp19a1a* promoter, namely, SBE1 (− 1372/−1364) and SBE2 (− 415/−407). Smad4 significantly suppressed the transcription of the *cyp19a1a* gene, and *cyp19a1a* transcriptional activity was significantly increased when SBE1 or SBE2 was mutated, but there was no difference between mut-SBE1 and mut-SBE2. These results suggest that Smad4 may act as a repressor to regulate the promoter activity of *cyp19a1a* via SBE1 and SBE2.

Previous studies have shown that FoxH1 regulates target genes as a transcriptional chaperone for Smad4 [49, 50]. FoxH1 in conjunction with Smad2/4 activated lim gene transcription [51]. Smad4 and FoxH1 are colocalized in the nuclei of RCC(Renal Cell Carcinom) cells, and Smad4 interacts with FoxH1 to repress estrogen receptor levels [52]. Further research proved that FoxH1 represses the transcriptional activity of the estrogen receptor [53]. In the present study, *smad4* and *foxh1* had consistent expression patterns, with a Pearson coefficient of 0.873. Smad4 and FoxH1 were colocalized in GCs and TCs. Compared with the overexpression of Smad4 alone, the co-overexpression of Smad4 and FoxH1 significantly reduced the promoter activity of *cyp19a1a*. These results indicated that Smad4 and FoxH1 negatively regulate *cyp19a1a* transcription individually and that their synergistic inhibitory effect is more significant.

Most previous studies on Smad4/FoxH1 functions have focused on embryogenesis, while little is known about the roles of Smad4/FoxH1 in reproduction. The ricefield eel is a protogynous hermaphrodite fish, and ovarian development is crucial for producing high-quality gametes. Aromatase plays an important role in ovarian development and female sex maintenance. The Smad family and Fox family transcription factors have been studied in mammals but rarely in bony fish. The results of this study provide a new perspective for the transcriptional regulation of aromatase in teleost fish.

### Perspectives and significance

*Monopterus albus* is a protogynous hermaphrodite fish that naturally undergoes a sex change from a functional female to a male through an intersexual phase of gonadal differentiation. The present study on the gonadal differentiation of *M. albus* provides novel insights from transcriptional factors Smad4 and FoxH1 to explore potential molecular mechanism of *cyp19a1a* transcription. In the future, gene editing technology will be used to explore the function of Smad4 and FoxH1 in the ovaries of *M. albus*, and granulosa cells of *M. albus* will be used as tool cells to study the transcriptional regulation of *cyp19a1a*. These results will provide a basic reference for fish reproductive endocrine.

## Conclusions

The expression trend of *smad4* and *foxh1* is similar during ovarian development, and the mRNA levels of *smad4* and *foxh1* change similarly after FSH incubation, and Pearson correlation analysis shows that *smad4* and *foxh1* are significantly positively correlated. Smad4, FoxH1 and Cyp19a1a colocalized in the oocyte, granulosa cells and theca cells. Smad4 was confirmed as a *cyp19a1a* transcriptional repressor and co-overexpression of Smad4 and FoxH1 significantly reduced *cyp19a1a* promoter activity. Thus, Smad4 may coordinate with FoxH1 to repress *cyp19a1a* transcription in CHO cells.

### Electronic supplementary material

Below is the link to the electronic supplementary material.

Supplementary Material 1

## Data Availability

All data supporting the findings of this study are available within the paper and its Supplementary Information.
