# Supplementary material for: Smad4 and FoxH1 potentially interact to regulate cyp19a1a promoter in the ovary of ricefield eel (Monopterus albus)
Source: Biol Sex Differ. 2024 Jul 30;15:60. doi: 10.1186/s13293-024-00636-w (PMC11290265; doi:10.1186/s13293-024-00636-w)
Supplement: Supplementary file 1 — Supplementary Material 1 [file 13293_2024_636_MOESM1_ESM.docx]

**Supplementary materials**

Table S1. Primers for cloning and mRNA expression analysis

| **Primer** | **Sequence (5′- 3′)** | **Product size (bp)** |
| --- | --- | --- |
| *smad4* F1 | TGGTCAGAAACATACAGCAAG | 892 bp |
| *smad4* R1 | CATCCATCTCAAAATAGGC |  |
| *smad4* F2 | TTCACCCCTAACATACCC | 899 bp |
| *smad4* R2 | TTCTCCACCCAAAACAGT |  |
| *smad4*-*qF* | TGGTGTCTCCTGGCATAG | 242 bp |
| *smad4*-*qR* | TGATGTTGAGGCAGAGGT |  |
| *smad2*-*qF* | AGCCCTCGCTGACAGTGGA | 230 bp |
| *smad2*-*qR* | TGGATGCCAGCCATACCG |  |
| *smad3*-*qF* | AGAAAGCCATCACCACCCAG | 186 bp |
| *smad3*-*qR* | GGAAGGCATACTCGCACAAC |  |
| *foxh1*-*qF* | CGATTACGCAGCGGGATT | 198 bp |
| *foxh1- qR* | GAGGCACTATGAGCAGAGGATG |  |
| *ef1α*-F | CGCTGCTGTTTCCTTCGTCC | 102 bp |
| *ef1α*-R | TTGCGTTCAATCTTCCATCCC |  |
| *rpl17*-F | GTTGTAGCGACGGAAAGGGAC | 160 bp |
| *rpl17*-R | GACTAAATCATGCAAGTCGAGGG |  |

F: forward primers; R: reverse primers.

Table S2 Mutation methods of Smad4 mutant plasmids

| **Plasmids** | **Mutation site (5**′**-3**′**)** | **Post-mutation site** |
| --- | --- | --- |
| Smad4-mut1 | TTGTCCAGA | ccagatgcc |
| Smad4-mut2 | TGTATAGA | caggctac |


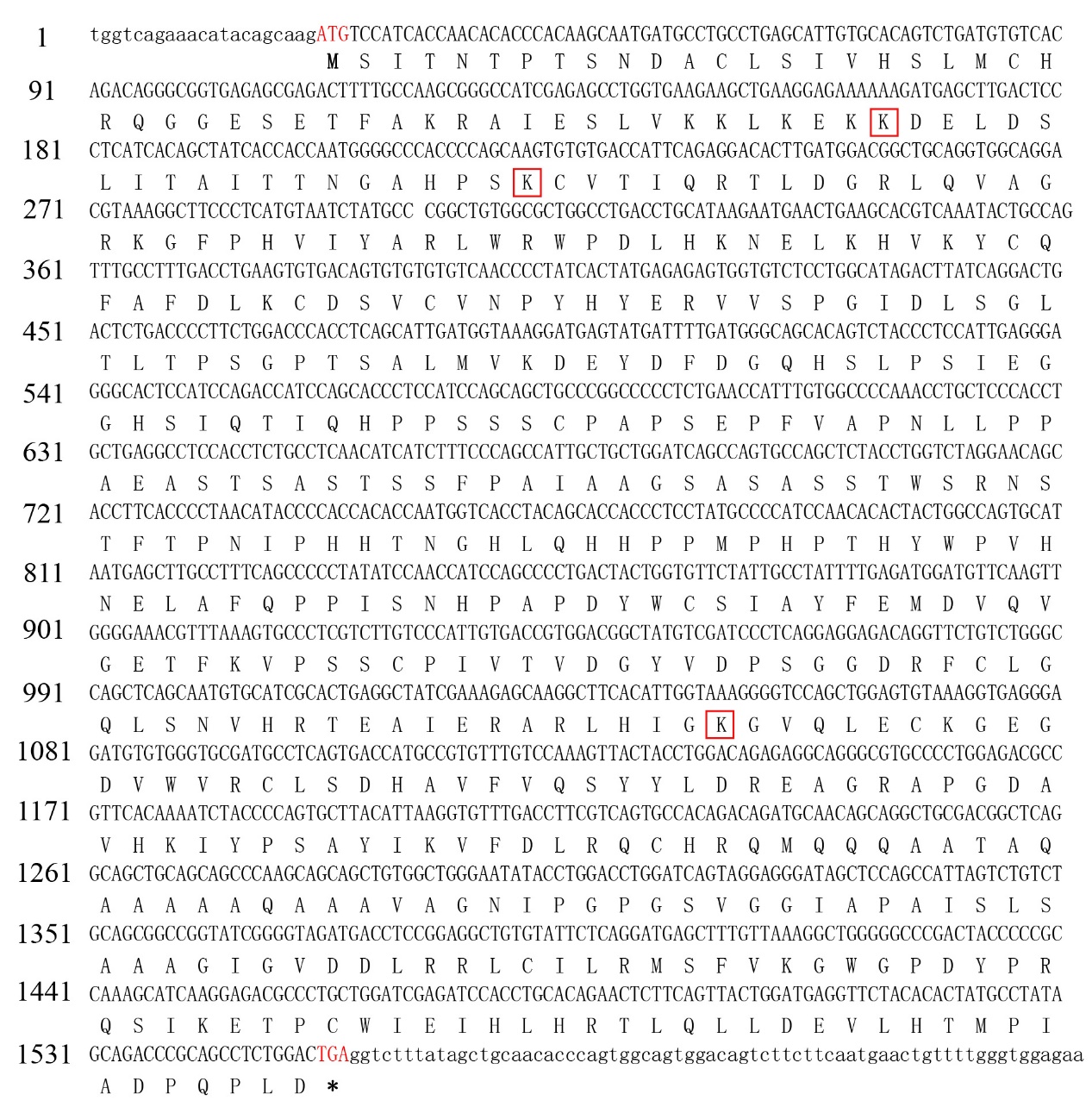


**Fig. S1 Nucleotide sequence information and amino acid sequence of the coding region of Smad4 in the ricefield eel *Monopterus albus*.** The untranslated regions and translated regions are indicated by lowercase letters and uppercase letters, respectively. The predicted L-acetylation sites are indicated with red boxes. The initiation codon (ATG) and stop codon (TAA) are marked in red. Asterisks (*) indicate the translation stop codon.
